# Correlations between Maternal, Breast Milk, and Infant Vitamin B12 Concentrations among Mother–Infant Dyads in Vancouver, Canada and Prey Veng, Cambodia: An Exploratory Analysis

**DOI:** 10.3390/nu9030270

**Published:** 2017-03-12

**Authors:** Philip Chebaya, Crystal D. Karakochuk, Kaitlin M. March, Nancy N. Chen, Rosemary A. Stamm, Hou Kroeun, Prak Sophonneary, Mam Borath, Setareh Shahab-Ferdows, Daniela Hampel, Susan I. Barr, Yvonne Lamers, Lisa A. Houghton, Lindsay H. Allen, Tim J. Green, Kyly C. Whitfield

**Affiliations:** 1 Food, Nutrition, and Health, The University of British Columbia, Vancouver, BC V6T 1Z4, Canada; philipchebaya@gmail.com (P.C.); crystal.karakochuk@alumni.ubc.ca (C.D.K.); marchkmr@gmail.com (K.M.M.); nnchen30@gmail.com (N.N.C.); susan.barr@ubc.ca (S.I.B.); yvonne.lamers@ubc.ca (Y.L.); kyly.whitfield@msvu.ca (K.C.W.); 2 BC Children’s Hospital Research Institute, Vancouver, BC V6H 3N1, Canada; 3 Department of Human Nutrition, University of Otago, Dunedin 9016, New Zealand; rosemary.stamm@otago.ac.nz (R.A.S.); lisa.houghton@otago.ac.nz (L.A.H.); 4 Helen Keller International—Cambodia Country Office, Phnom Penh 12301, Cambodia; hkroeun@hki.org; 5 National Nutrition Programme, Maternal and Child Health Centre, Ministry of Health, Phnom Penh 12202, Cambodia; sophonprak@gmail.com; 6 National Sub-Committee for Food Fortification, Ministry of Planning, Phnom Penh 12000, Cambodia; borathmam@yahoo.com; 7 US Department of Agriculture, ARS Western Human Nutrition Research Centre, University of California, Davis, CA 95616, USA; setti.shahab-ferdows@ars.usda.gov (S.S.-F.); daniela.hampel@ars.usda.gov (D.H.); lindsay.allen@ars.usda.gov (L.H.A.); 8 Department of Nutrition, University of California, Davis, CA 95616, USA; 9 South Australia Health and Medical Research Institute, Adelaide, South Australia 5000, Australia; 10 Department of Applied Human Nutrition, Mount Saint Vincent University, Halifax, NS B3M 2J6, Canada

**Keywords:** vitamin B12 (cobalamin), lactation, human milk, Canada, Cambodia

## Abstract

Vitamin B12 plays an essential role in fetal and infant development. In regions where animal source food consumption is low and perinatal supplementation is uncommon, infants are at risk of vitamin B12 deficiency. In this secondary analysis, we measured total vitamin B12 concentrations in maternal and infant serum/plasma and breast milk among two samples of mother–infant dyads in Canada (assessed at 8 weeks post-partum) and in Cambodia (assessed between 3–27 weeks post-partum). Canadian mothers (*n* = 124) consumed a daily vitamin B12-containing multiple micronutrient supplement throughout pregnancy and lactation; Cambodian mothers (*n* = 69) were unsupplemented. The maternal, milk, and infant total vitamin B12 concentrations (as geometric means (95% CI) in pmol/L) were as follows: in Canada, 698 (648,747), 452 (400, 504), and 506 (459, 552); in Cambodia, 620 (552, 687), 317 (256, 378), and 357 (312, 402). The majority of participants were vitamin B12 sufficient (serum/plasma total B12 > 221 pmol/L): 99% and 97% of mothers and 94% and 84% of infants in Canada and Cambodia, respectively. Among the Canadians, maternal, milk, and infant vitamin B12 were all correlated (*p* < 0.05); only maternal and infant vitamin B12 were correlated among the Cambodians (*p* < 0.001).

## 1. Introduction

Vitamin B12 is found only in animal-source or fortified foods [1], so deficiency is most common in those who consume vegan diets [2] or in populations in low- and middle-income countries where animal foods are not available or affordable [3]. Maternal vitamin B12 status in pregnancy strongly influences infant vitamin B12 status [4]; breast milk vitamin B12 content is reduced when maternal vitamin B12 status is poor [5]. Since exclusive breastfeeding is recommended for the first 6 months of life [6], infants rely solely on their stores and breast milk for vitamin B12 during this time [2]. Attaining adequate vitamin B12 status in infancy—a time of rapid growth and development—is essential for normal cognitive development in infants [7]. Deficiency, which typically presents in infants between 4 and 10 months but may appear within months of birth, can lead to failure to thrive, developmental regression [2], and severe neuropathy [8]. 

There is currently little published data describing blood and milk vitamin B12 concentrations of purportedly well-nourished mother–infant dyads [4]. For instance, there is currently no agreed-upon cut-off for vitamin B12 deficiency in breast milk; although a cut-off of <362 pmol/L was used to define deficiency previously, it was developed over 25 years ago using older laboratory methods that may have incorrectly measured vitamin B12 analogues, and included only 19 milk samples [9,10]. Greibe et al. measured plasma vitamin B12 concentrations among 60 Danish mother–infant dyads, and breast milk vitamin B12 among a subset of 25 mothers at 2 weeks, 4 months, and 9 months postpartum [11]. The majority of those women were consuming daily vitamin B12-containing multivitamin supplements. Moderate correlations (*r =* 0.27 to 0.52) were found between maternal and infant vitamin B12 concentrations at all three time-points. Milk vitamin B12 concentrations, however, fluctuated considerably throughout lactation, and were only significantly correlated with maternal and infant plasma vitamin B12 concentrations at 4 months postpartum [11]. In contrast, the authors of an earlier study reported that breast milk B12 concentrations remained relatively constant after falling from their highest levels in colostrum [12]. 

With the relatively limited data currently available on perinatal vitamin B12 status, we used secondary data from two recent randomized control studies [13,14] to assess the vitamin B12 status of mothers, their breast milk, and their infants in two regions with differing perinatal supplementation and dietary patterns: Canada and Cambodia. We also explored associations between vitamin B12 concentrations in maternal blood, breast milk, and infant blood samples.

## 2. Materials and Methods

### 2.1. Study Populations and Biological Sample Collection

This was a secondary analysis of two studies conducted with exclusively breastfeeding mother–infant dyads in Vancouver, British Columbia, Canada (Canada sample), and Prey Veng province, Cambodia (Cambodian sample). In both samples, a short demographic questionnaire was conducted among participants to gather information such as age, antenatal care, and socioeconomic status; the questionnaire was self-administered in Canada, and interviewer-administered in Cambodia.

#### 2.1.1. Canadian Sample

Participants in the Canadian sample were enrolled as part of a randomized control trial from June 2010 through March 2013, investigating the efficacy of three doses of maternal perinatal vitamin D to improve infant 25-hydroxyvitamin D concentrations at 8 weeks postpartum; results are published elsewhere [13]. Briefly, healthy pregnant women 18–42 years with a low-risk singleton pregnancy in Vancouver, British Columbia were recruited using convenience sampling. Women in this study were randomized to consume an identical daily perinatal multivitamin supplement containing 12 µg vitamin B12 starting at 13 to 22 weeks gestation through to 8 weeks post-partum, and a second daily supplement that contained 10, 25, or 50 µg vitamin D3. This study was approved by the British Columbia Children’s and Women’s Clinical Research Ethics Board (H13-01971). 

At 8 weeks postpartum, non-fasting venous blood samples were collected from mothers and infants into trace element-free evacuated tubes (Vacutainer, Becton Dickinson, Mississauga, ON, Canada). The samples were allowed to clot at room temperature for 30 min, centrifuged (2000× *g* for 10 min at 4 °C), and then the serum was removed, aliquoted, and stored at −80 °C until analysis. Breast milk samples were collected either at the clinic during the phlebotomy visit or by women themselves the morning of the phlebotomy visit. Regardless of collection site, milk from one full breast expression was collected using an electric breast pump (the woman’s own, or Swing breast pump, Medela), more than two hours after the previous feeding. If women collected milk themselves at home, the sample was stored in their refrigerator until the phlebotomy visit (<10 h). At the clinic, the volume of the milk sample was recorded, and then mixed thoroughly, aliquoted, and stored at −80 °C until analysis.

#### 2.1.2. Cambodian Sample

Participants in the Cambodian sample were recruited as part of a randomized control trial designed to test the efficacy of maternal ad libitum consumption of thiamin-fortified or control (non-thiamin fortified) fish sauce to improve maternal and infant erythrocyte thiamin diphosphate concentrations and breast milk thiamin concentrations [14]. Healthy women 18–45 years with a low-risk singleton pregnancy residing in Prey Veng province, Cambodia, were recruited between 3–8 months gestation (self-report), and consumed fish sauce for 6 months as part of this study. All women received 90 iron-folic acid supplements during pregnancy; no participants consumed vitamin B12-containing supplements. The National Ethics Committee for Health Research in Cambodia approved this study (0245 NECHR; 386 NECHR). 

Between 3–27 weeks postpartum (in April 2015), trained phlebotomists collected non-fasting venous blood samples from mothers and their infants into EDTA-coated tubes (Vacutainer, Becton Dickinson). The samples were placed immediately on ice, and were transported to the National Institute of Public Health (NIPH) laboratory in Phnom Penh within 5 h of collection. Samples were centrifuged (3000 rpm for 15 min at 4 °C), and plasma removed, aliquoted, and stored at −80 °C. Breast milk samples were collected during the phlebotomy visit using a procedure identical to that in the Canadian study. Breast milk samples were placed on ice and transported to the NIPH laboratory with the blood samples. Total milk volume was recorded, then each sample was mixed thoroughly, aliquoted into amber cryovials, and stored at −80 °C. Both blood and breast milk samples were shipped on dry ice to The University of British Columbia in Vancouver, Canada, for storage at −80 °C until being shipped to other laboratories for analysis.

### 2.2. Biological Sample Analysis

Serum/plasma samples were shipped on dry ice to the University of Otago in Dunedin, New Zealand for analysis in the Houghton Lab. Total vitamin B12 concentration was measured using an electrochemiluminescence immunoassay (Roche Diagnostics) on an Elecsys 2010 (Roche, New Zealand). Control samples fell within the recommended manufacturer detection range of 22–1495 pmol/L; inter-assay coefficient of variance of a pooled serum sample with a known vitamin B12 concentration within the detection range was 8.3% (*n* = 13). Total vitamin B12 concentration was measured in serum samples from 124 Canadian mothers and 102 infants; and in plasma samples from 69 Cambodian mothers and 50 infants. 

Human milk vitamin B12 concentrations were measured using chemiluminescence on an IMMULITE automated analyzer in the Allen Lab, US Department of Agriculture/ARS Western Human Nutrition Research Centre, Davis; methods reported in detail elsewhere [15]. Milk samples were boiled in dithiothreitol and potassium cyanide to release protein-bound vitamin B12 before analysis. Breast milk vitamin B12 concentration was measured in 109 Canadian and 59 Cambodian samples.

### 2.3. Statistical Analysis

Only those mothers who self-reported exclusive breastfeeding were included in the analysis. Demographic characteristics are expressed as mean (95% CI) and *n* (%) for continuous and categorical variables, respectively, unless otherwise noted. Concentrations of vitamin B12 were not normally distributed in either sample for any of the biological specimens (*p* < 0.05 for Shapiro–Wilk test of normality), and thus were transformed using the natural log for analyses and back-transformed and expressed as geometric mean (95% CI). Independent sample *t*-tests were used to assess differences in maternal age, parity, and maternal, milk, and infant total vitamin B12 concentrations, and chi-square tests were used to assess differences in educational attainment and household income between the Canadian and Cambodian samples. Pearson’s bivariate correlations (Canada) and partial correlations (Cambodia, controlled for infant age) were used to determine correlations between maternal and infant total vitamin B12 concentrations, and between milk vitamin B12 concentrations and both maternal and infant total vitamin B12 concentrations.

All infants in the Canadian sample were evaluated at 2 months ± 1 week of age; infants in the Cambodian sample ranged from 3 to 27 weeks of age, with a mean (SD) age at sample collection of 15 (7) weeks. Therefore, linear regression models were run in the Cambodian sample to assess the effect of infant age on maternal, infant, and milk vitamin B12 concentrations. 

Vitamin B12 deficiency is defined here as serum/plasma total vitamin B12 concentration < 148 pmol/L, and marginal deficiency between 148–221 pmol/L [1]. Since there are no cut-offs specifically designed for use in infants, the same cut-offs were employed for both mothers and infants. Chi-square tests were used to determine if there was a difference in the prevalence of serum/plasma vitamin B12 deficiency between groups.

All analyses were performed on SPSS for Macintosh version 23.0 (IBM Corp, Armonk, NY, USA), with a significance level of *p* < 0.05.

## 3. Results

All Cambodian mothers were Khmer; in Canada, the majority of mothers (*n =* 97; 79%) were of European descent (7% were Chinese, and 14% were of 12 other ethnicities). Canadian mothers were older than the Cambodian mothers: mean (95% CI) age was 37 (36, 38) and 26 (25, 27) years, respectively (*p* < 0.001). The Canadian mothers also had greater educational attainment and annual household income than the Cambodian participants (*p* < 0.001). Approximately half of mothers—53% in Canada and 54% in Cambodia—were pregnant for the first time (*p* = 0.66). 

**Figure 1:**
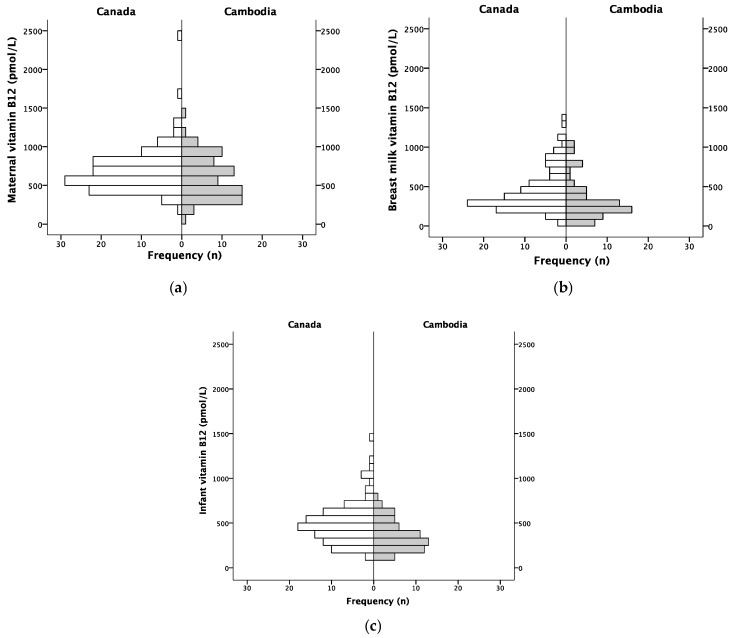
Histograms of (**a**) maternal total vitamin B12 concentrations (pmol/L), (**b**) human milk total vitamin B12 concentrations (pmol/L), and (**c**) infant total vitamin B12 concentrations (pmol/L) among the Canadian (white bars) and Cambodian (shaded bars) samples.

Biochemical vitamin B12 status is presented in Table 1. Maternal and infant serum/plasma and breast milk total vitamin B12 concentrations were all significantly higher among the Canadian as compared to the Cambodian sample (*p* < 0.05), although the distributions of mothers’ and infants’ B12 status did not differ between groups. Histograms showcasing the maternal, milk, and infant total vitamin B12 concentrations of the Canadian and Cambodian samples are shown in Figure 1. 

Correlations between maternal, infant, and breast milk total vitamin B12 concentrations are shown in Table 2. In the Canadian sample, all three variables were significantly correlated with one another (*p* < 0.05). In the Cambodian sample, however, only maternal and infant total vitamin B12 concentrations were correlated (*p* < 0.001); breast milk total vitamin B12 concentrations were not correlated with either maternal (*p* = 0.43) or infant (*p =* 0.64) total vitamin B12 concentrations. 

Unlike in Canada, where all samples were collected at 2 months post-partum, data and sample collection in Cambodia took place when infants were aged between 3 and 27 weeks, or mean (SD) of 15 (7) weeks postpartum. We used linear regression models to examine the association between infant age and plasma and milk vitamin B12 concentrations among the Cambodian sample, and found that none of these vitamin B12 measurements were significantly correlated with infant age (Table 3). While infant age was borderline inversely associated with milk vitamin B12 concentrations (*p* = 0.05), age would have accounted for <5% of the variance in milk B12 concentrations. Table 4 displays the prevalence of vitamin B12 inadequacy among the Cambodian sample by infant age; 8 weeks was used as a cut-off to align with the Canadian sample. Again, infant age appeared to influence only milk vitamin B12 concentrations, with older infants (>8 weeks) tending to have lower mean concentrations.

## 4. Discussion

Milk vitamin B12 concentrations reported in the literature vary considerably. Greibe and colleagues reported median concentrations of vitamin B12 in the hindmilk of 25 well-nourished Danish mothers of 760, 290, and 440 pmol/L at 2 weeks, 4 months, and 9 months postpartum, respectively [11], indicating variability with lactation stage. In this study, we found higher total vitamin B12 concentrations in the milk of Cambodian mothers ≤ 8 weeks postpartum (*n =* 13) compared with >8 weeks postpartum (*n =* 46) (427 versus 286 pmol/L, respectively), however this was not statistically significant (*p =* 0.051; Table 4). Regardless, these results are difficult to compare due to the low sample sizes, differing time-points for milk collection, and because we collected a full milk expression, while only hindmilk was collected in the Danish study.

As noted earlier, a milk vitamin B12 deficiency cut-off of <362 pmol/L exists [9,10], but is not commonly employed due to potential overestimation of inadequacy. Nearly all mothers in both Canada and Cambodia (≥97%) had adequate vitamin B12 status (>221 pmol/L), as did their infants—94% and 84%, respectively. However, the vitamin B12 status of breast milk was below the cut-off of 362 pmol/L [9,10] among 50% and 75% of Canadian and Cambodian mothers, respectively (data not shown). The high prevalence of adequate vitamin B12 status among infants despite less than adequate breast milk vitamin B12 levels highlights the necessity for more research to better define deficient vitamin B12 breast milk levels given current laboratory methods. 

Consistent with previous studies, we found significant correlations between maternal and infant serum/plasma total vitamin B12 concentrations in both the Canadian and Cambodian samples. For example, Greibe et al. identified significant correlations (*p* < 0.05) between maternal and infant vitamin B12 concentrations at three different time-points among Danish infants aged 2 weeks (*r* = 0.52), 4 months (*r* = 0.47), and 9 months (*r* = 0.29) [11]. Similarly, significant correlations between maternal and infant plasma vitamin B12 were reported among Malawian dyads (*n =* 521) in the Breastfeeding, Antiretroviral, and Nutrition (BAN) study—*r* = 0.42 and 0.32 at 2 or 6 weeks and 24 weeks, respectively [16]. 

In our study, we found that milk vitamin B12 concentration was not significantly correlated with infant total vitamin B12 in the Cambodian sample; however, at 2 months post-partum, milk was significantly but weakly correlated with infant serum vitamin B12 concentrations in the Canadian sample (*r* = 0.108, *p* < 0.001; see Table 2). The vitamin B12 content of milk is impacted by maternal dietary vitamin B12 intake and depletion [5]; however, the association between milk vitamin B12 and infant vitamin B12 status is not consistent. Greibe et al. found that milk was only significantly correlated with infant plasma vitamin B12 concentrations at 4 months postpartum (*r* = 0.58; *p* = 0.005) but not 2 weeks or 9 months [11]. In a Guatemalan study of 113 dyads at 3 months postpartum, the authors reported that milk vitamin B12 concentrations were inversely associated with infant vitamin B12 status (*r* = −0.22; *p* < 0.05) as assessed by urinary methylmalonic acid [10]. The BAN study in Malawi reported significant correlations between maternal plasma, breast milk, and infant plasma vitamin B12 concentrations at both 2 or 6 and 24 weeks, but speculated that maternal vitamin B12 status in pregnancy is likely a more important predictor of infant status than milk concentrations [16]. It may be that maternal vitamin B12 status during pregnancy is a better indicator of infant vitamin B12 adequacy in the first year of life than milk vitamin B12 concentrations, given that infants of well-nourished mothers are born with ~25 μg of vitamin B12 stores [17], which should protect the infant from inadequacy to 12 months of age regardless of milk vitamin B12 content.

Maternal and infant serum/plasma and breast milk total vitamin B12 concentrations were significantly higher among the Canadian sample compared to the Cambodian sample (*p* < 0.05). These differences were not unexpected, because the Canadian mothers were consuming a daily vitamin B12-containing perinatal supplement providing >400% of the recommended dietary allowance for lactation [18]. Although all Cambodian mothers were consuming fish sauce as part of the original randomized control trial [14], this is not a significant source of dietary vitamin B12; American fish sauce provides only ~0.05 μg vitamin B12 per 10 mL fish sauce [19] as per international CODEX standards [20]. The lack of vitamin B12 deficiency in Cambodia is consistent with the most recent Cambodian Demographic and Health Survey (2014), which reported only 1% of mothers (*n =* 731) were vitamin B12 deficient (<150 pmol/L) [21]. 

This study has several strengths, namely the collection of three biological samples from mother–infant dyads at the same time-point (mother and infant blood, and breast milk). In addition, a full breast expression was collected, and samples were collected from the majority of women first thing in the morning, which helps to limit intra-participant diurnal variation, although this is not a major concern with vitamin B12 [22]. We also employed current laboratory methods to assess milk vitamin B12 [15]; older methods may have incorrectly measured vitamin B12 analogues. However, as a secondary analysis, this study has several shortfalls. It would have been ideal to measure at least one direct and one functional indicator for vitamin B12 status in addition to total vitamin B12 concentration. However, due to a lack of blood samples and funding, we were unable to measure methylmalonic acid or total homocysteine. We selected total vitamin B12 concentration as our indicator because it is the most commonly employed and inexpensive direct biomarker [23]. The lack of longitudinal data is a limitation, especially given that vitamin B12 levels appear to fluctuate throughout lactation. In addition, breast milk values from Cambodia cannot be directly compared against those from Canada because they were not collected at the same time; while early literature indicates that breast milk B12 concentrations remain relatively constant after falling from their highest levels in colostrum [12], a more recent study reported fluctuation throughout lactation [11]. Our analyses included only exclusively breastfeeding dyads, and therefore it is less relevant for comparison with children consuming breast milk substitutes or those who have started consuming complementary foods. Dietary data were not collected, limiting our ability to generalize these findings to populations with known high- or low-vitamin B12 intakes. Anthropometric measures were not assessed at the time of blood and milk collection in either group. In addition, these samples were not representative, limiting generalizability. 

## 5. Conclusions

The vast majority of both Canadian and Cambodian mothers (≥97%) and infants (≥84%) had sufficient vitamin B12 status (>221 pmol/L), however maternal, infant, and milk total vitamin B12 concentrations were significantly higher among the Canadian sample compared to the Cambodians. In the Canadian sample, maternal, milk, and infant vitamin B12 concentrations all significantly correlated, while only maternal and infant plasma vitamin B12 concentrations were correlated in the Cambodian sample. The wider age range among Cambodian infants (3 to 27 weeks) allowed for the exploration of associations in infant age revealing a borderline significant inverse relationship with infant age and infant B12 status. The vast majority of both mothers (≥97%) and infants (≥84%) in both groups had sufficient vitamin B12 status (>221 pmol/L). 

## Figures and Tables

**Table 1:** Vitamin B12 status among mothers and their infants in Vancouver, Canada, and Prey Veng province, Cambodia ^1,2^.

Vitamin B12 Marker	*n*	Vancouver, Canada	*n*	Prey Veng, Cambodia	*p* Value
Maternal vitamin B12	124	698 (648, 747)	69	620 (552, 687)	0.009
Deficient (<148)		-		1 (~1%)	0.37
Marginal (≥148 to 221)		1 (1%)		1 (~1%)	
Sufficient (≥221)		123 (99%)		67 (97%)	
Infant vitamin B12	102	506 (459, 552)	50	357 (312, 402)	<0.001
Deficient (<148)		2 (2%)		4 (8%)	0.10
Marginal (≥148 to 221)		4 (4%)		4 (8%)	
Sufficient (≥221)		96 (94%)		42 (84%)	
Breast milk vitamin B12 ^3^	109	452 (400, 504)	59	317 (256, 378)	<0.001

^1^ Serum (Canada) and plasma (Cambodia) total vitamin B12 (pmol/L), and breast milk vitamin B12 (pmol/L) expressed as geometric mean (95% CI); vitamin B12 status expressed as *n* (%); ^2^ Independent samples *t*-tests and chi-square tests were employed to assess differences between samples from Canadians and Cambodians of vitamin B12 concentrations and adequacy status categories, respectively; ^3^ Breast milk samples collected at 2 months postpartum among Canadian women, and between 3–27 weeks (mean (SD) was 15 (7) weeks) postpartum in the Cambodian sample.

**Table 2:** Correlations between maternal, infant, and milk total vitamin B12 concentrations among samples of mother–infant dyads in Canada and Cambodia.

Vitamin B12 marker	*n*	Correlation ^1^	*p* Value
Maternal vitamin B12 and breast milk vitamin B12			
Vancouver, Canada	109	0.498	<0.001
Prey Veng, Cambodia	59	0.105	0.43
Maternal vitamin B12 and infant vitamin B12			
Vancouver, Canada	102	0.208	0.04
Prey Veng, Cambodia	49	0.562	<0.001
Breast milk vitamin B12 and infant vitamin B12			
Vancouver, Canada	88	0.370	<0.001
Prey Veng, Cambodia	45	0.073	0.64

^1^ Pearson’s bivariate correlations were used for the Canadian sample; partial correlations controlling for infant age were used in the Cambodian sample.

**Table 3:** Linear regression models of the association between infant age and plasma and milk vitamin B12 concentrations among the Cambodian sample.

Vitamin B12 Marker	Adjusted *R*^2^	Unstandardized		Standardized β	*p* Value
		β	95% CI		
Maternal plasma vitamin B12	0.038	−0.015	−0.031, 0.001	−0.229	0.06
Milk vitamin B12	0.049	−0.028	−0.056, 0.000	−0.256	0.05
Infant plasma vitamin B12	0.021	−0.013	−0.032, 0.005	−0.202	0.16

**Table 4:** Total vitamin B12 concentrations and prevalence of vitamin B12 sufficiency by infant age (older or younger than 8 weeks) among the Cambodian sample ^1^.

Vitamin B12 Marker	*n*	Infant ≤ 8 weeks	*n*	Infant > 8 weeks	*p* Value
Maternal plasma vitamin B12	18	634 (464, 803)	51	615 (541, 688)	0.85
Deficient (<148)		-		-	0.73
Marginal (≥148 to 221)		-		1 (2%)	
Sufficient (≥221)		18 (100%)		50 (98%)	
Infant plasma vitamin B12	12	343 (253, 433)	38	361 (307, 415)	0.93
Deficient (<148)		-		4 (10%)	0.50
Marginal (≥148 to 221)		1 (7%)		3 (8%)	
Sufficient (≥221)		11 (93%)		31 (82%)	
Breast milk vitamin B12 ^2^	13	427 (266, 588)	46	286 (222, 351)	0.051

^1^ Total plasma vitamin B12 (pmol/L), and breast milk vitamin B12 (pmol/L) expressed as geometric mean (95% CI); vitamin B12 status expressed as *n* (%). Independent samples *t*-tests were employed to assess differences in vitamin B12 concentrations, and chi-square tests were used to assess differences in the prevalence of vitamin B12 adequacy between infant age groups; ^2^ Breast milk samples were collected between 3–27 weeks (mean (SD) was 15 (7) weeks) postpartum.
